# The separate and combined effects of MHC genotype, parasite clone, and host gender on the course of malaria in mice

**DOI:** 10.1186/1471-2156-7-55

**Published:** 2006-11-21

**Authors:** Claus Wedekind, Mirjam Walker, Tom J Little

**Affiliations:** 1 Department of Ecology and Evolution, University of Lausanne, Biophore, 1015 Lausanne, Switzerland; 2 Institute of Evolutionary Biology, School of Biological Sciences, University of Edinburgh, West Mains Road, Edinburgh EH9 3JT, Scotland, UK; 3 Institute of Zoo and Wildlife Biology, 10252 Berlin, Germany; 4 Natural History Museum Bern, 3005 Bern, Switzerland; 5 ZLB Behring AG, Wankdorfstr. 10, 3022 Bern, Switzerland

## Abstract

**Background:**

The link between host MHC (major histocompatibility complex) genotype and malaria is largely based on correlative data with little or no experimental control of potential confounding factors. We used an experimental mouse model to test for main effects of MHC-haplotypes, MHC heterozygosity, and MHC × parasite clone interactions. We experimentally infected MHC-congenic mice (F2 segregants, homo- and heterozygotes, males and females) with one of two clones of *Plasmodium chabaudi *and recorded disease progression.

**Results:**

We found that MHC haplotype and parasite clone each have a significant influence on the course of the disease, but there was no significant host genotype by parasite genotype interaction. We found no evidence for overdominance nor any other sort of heterozygote advantage or disadvantage.

**Conclusion:**

When tested under experimental conditions, variation in the MHC can significantly influence the course of malaria. However, MHC heterozygote advantage through overdominance or dominance of resistance cannot be assumed in the case of single-strain infections. Future studies might focus on the interaction between MHC heterozygosity and multiple-clone infections.

## Background

The link between the MHC (major histocompatibility complex) and malaria [1,2] is a widely cited example of a link between host genes and the course of a disease. However, for humans, such links cannot be studied under controlled experimental conditions. Therefore, the significance of the MHC for disease severity is still not fully understood [3-12].

The mouse model offers the possibility of studying potential MHC effects both under experimental conditions *and *in congenic strains, i.e. in strains that differ only in the MHC region but are identical on the rest of the genome [13-16]. However, even when working with MHC congenic strains, potential confounding effects remain. This include, firstly, host age which may influence pathogen susceptibility and should therefore not vary with MHC genotype. Secondly, maternal environmental effects are known to affect offspring size, number, and general vigour [17,18]. Such maternal effects could explain why some congenic strains produce different olfactory signals only in parental strains but not in F_2 _segregants [19], or why congenic strains sometimes differ in behavior [20]. Lastly, MHC-congenic lines may differ with respect to the mutation load on their background genes, as there is at least one example of different mortalities during the first days of development before blastocyst formation (as tested under controlled *in vitro *conditions in Wedekind *et al*. [21]). As variation in age, maternal effects or mutation load could interact with pathogen susceptibilities [22], studies which aim to isolate the effects of particular loci must use breeding designs that randomize all background effects [22-24]. F_2 _segregants bred and reared under controlled conditions can account for such possibly confounding effects [22,25-27].

We studied F_2 _segregants of MHC congenic strains during experimental exposure to two clones of *Plasmodium chabaudi (*such infections share many similarities to *P. falciparum *infections in humans [28,29]). We first addressed the following questions: (i) Do MHC genotypes differ in their response to malaria when tested under experimental conditions? (ii) Is there a significant interaction between the MHC-haplotypes and the malaria clones on disease severity? (iii) Is there an MHC heterozygote advantage in our example of single-clone infections?

These questions were previously addressed in mice with segregaring H-2^a ^and H-2^b ^haplotypes [30]. H-2^a ^homozygotes turned out to be more susceptible to both malaria clones than H-2^b ^homozygotes, and H-2^ab ^heterozygotes did worse than expected from the average response of the homozygotes. Here, we tested additional MHC genotypes, H-2^k^, H-2^ak^, and H-2^bk^, with the same methods and the same pathogen strains as in our previous study. This expanded analysis allowed us to address some further questions: (iv) In general, are the previously observed separate and combined effects of host MHC, host gender, pathogen clone, and the time course of the disease symptoms repeatable when tested on other host genotypes? (v) In particular, does the H-2^a ^haplotype again cause higher susceptibilties than the H-2^b ^haplotype if combined with another haplotype (the H-2^k^)? (vi) Can we add two more examples of heterozygotes doing worse than expected from the average response of their respective homozygotes?

## Results

### The separate and combined effects of MHC, host gender, and parasite clone

We first concentrate on MHC effects in the two heterozygous genotypes. The fully-factorial experimental design allowed study of the separate and combined impact of MHC type (H-2^ak^, H-2^bk^), host gender (two sexes), and parasite clone ("AS" and "CW"). We tracked the time course of the disease in daily weight measurements, and in repeated parasitemia counts and blood cell counts. The resulting repeated measures analyses of variances (ANOVAs) are summarized in Table 1 and discussed below. Variation in age was minimised by synchronized breeding and excluding one exceptionally young mouse from the analyses. When the above ANOVA model was run on age as dependent variable (instead of any of the disease parameters), none of the factors are significantly linked to age, i.e. age did not significantly vary with MHC-type, gender, or pathogen clone. Body weights and blood cell densities at the day of exposure or one day later were also not significantly different between the experimental groups, except that, as expected [30,31], males were initially heavier than females (t = 11.2, p < 0.0001) and had lower blood cell densities (t = 3.4, p = 0.002).

**Table 1:** The effect of host MHC, parasite clone and host gender on the time course of disease symptoms.

	Parasitemia			Blood cell counts			Body weight change		
	F	d.f.	P	F	d.f.	P	F	d.f.	P
Between subjects									
Host MHC	11.1	1, 38	**0.002**	3.9	1, 37	**0.057**	0.1	1, 37	0.79
*Plasmodium *clone	<0.1	1, 38	**0.95**	0.9	1, 37	0.35	0.4	1, 37	0.52
Host gender	0.4	1, 38	0.51	3.1	1, 37	**0.09**	<0.1	1, 37	1.0
									
MHC × clone	0.5	1, 38	0.50	1.5	1, 37	0.24	2.7	1, 37	0.11
MHC × gender	<0.1	1, 38	0.86	0.2	1, 37	0.63	<0.1	1, 37	0.91
Clone × gender	2.9	1, 38	**0.10**	8.6	1, 37	**0.006**	0.3	1, 37	0.62
MHC × clone × gender	0.1	1, 38	0.79	3.0	1, 37	0.09	4.8	1, 37	0.036
									
Within subjects (repeated measurements on individual mice)									
Time	79.1	5, 34	**<0.0001**	88.4	10, 28	**<0.0001**	15.8	18, 20	**<0.0001**
									
Time × MHC	3.3	5, 34	**0.016**	0.7	10, 28	0.74	1.0	18, 20	0.53
Time × clone	4.5	5, 34	**0.003**	4.3	10, 28	**0.001**	3.6	18, 20	**0.003**
Time × gender	0.2	5, 34	0.95	3.2	10, 28	0.008	2.9	18, 20	**0.013**
									
Time × MHC × clone	0.7	5, 34	0.64	2.2	10, 28	0.051	1.5	18, 20	0.19
Time × MHC × gender	0.5	5, 34	0.74	1.9	10, 28	0.09	0.6	18, 20	0.86
Time × clone × gender	1.3	5, 34	0.28	1.4	10, 28	0.22	2.3	18, 20	**0.037**
Time × MHC × clone × gender	1.2	5, 34	0.34	1.6	10, 28	0.16	1.5	18, 20	0.18

The experiment was designed for a fully-factorial repeated measures analysis of variance (ANOVA), incorporating the fixed effect factors "host MHC", "host gender", and "*Plasmodium *clone", with repeated measures of the following dependent variable: parasitemia (6 measurements from day 4–14), blood cell counts (11 measurements from day 1–22), and body weight (19 measurements from day 4–22) given as differences to the weight at day 0. For within-subject analyses we used the multivariate F-tests. One H-2^ak ^male had to be euthanized at day 15 and could therefore only be included in the analysis on parasitemia. P-values that were = 0.05 in analogous tests on other H-2 genotypes [30] are marked in bold.

**Figure 1:**
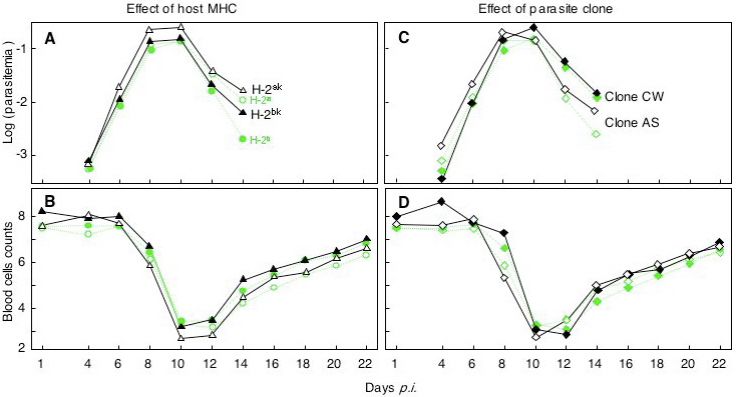
**The effects of MHC and parasite clone on the course of disease symptoms**. The mean parasitemia (log_10_-transformed) and the mean blood cell number (*10^9^)/ml blood for MHC genotypes H-2^ak ^(open triangles) and H-2^bk ^(closed triangles), or pathogen clone AS (open diamonds) and clone CW (closed diamonds) are given over the course of the disease. Black symbols and lines refer to the data collected in this study on H-2^ak ^and H-2^bk ^mice, the green symbols and hatched lines refer to previously published results on homozygous H-2^a ^and H-2^b ^mice for comparison (Fig. 1 in [30]. See Table 1 for statistics.

Consistent with previous studies [30,32,33], mean parasitemia rose dramatically during the first 10 days post infection (*p.i.*), and minimal blood cell counts and body weights were reached on average at day 10 and 12 *p.i.*, respectively (Fig. 1). At that stage, the mice had lost on average of 4.7% (SE = 1.0) of their initial body weight and 65.8% (SE = 1.8) of their initial red blood cells. All but two mice survived the acute phase of the infection and recovered as parasitemia declined over the next few days (following UK Home Office regulations, one H-2^bk ^male was euthanized at day 12 *p.i*. and one H-2^ak ^male at day 15 *p.i*.).

Hosts' MHC genotype had a strong effect on overall parasitemia (p = 0.002, Table 1) and the time course of parasitemia (p = 0.016; see also Figure 1a). The MHC also tended to influence blood cell counts (p = 0.057) but did not significantly affect body weight change (p = 0.79, Table 1). The H-2^ak ^genotype was more susceptible than the H-2^bk ^type (Fig. 1a, b).

The two parasite clones mainly differed in the time course of their effects on hosts, with clone AS having its peak parasitemia earlier than clone CW (p = 0.003 for clone × time term, Table 1, Fig. 1c). This corresponds with a similar pattern in the time course of the blood cell counts (p = 0.001 for clone × time, Table 1, Fig. 1d) and a corresponding change in host weight over time (p = 0.003 for clone × time, Table 1). The two sexes varied over time in their blood cell counts (p = 0.008, Table 1) and body weight change (p = 0.013, Table 1), and they reacted differently to the parasite clones (significant clone × gender, time × clone × gender, and MHC × clone × gender interactions, Table 1).The two clones did not significantly differ in overall parasitemia, blood cell counts, or weight differences (p always > 0.35, Table 1). Moreover, there was no significant interaction between parasite clone and MHC genotype in any of the disease symptoms (MHC × clone, Table 1).

### Comparing homozygotes to heterozygotes

To the above data on H-2^ak ^and H-2^bk ^heterozygotes, we added data from H-2^k ^homozygotes, as well as data from our previous study on H-2^a^, H-2^b^, and H-2^ab ^mice [30] and thus offer an overall analysis that compares the effects of six different MHC genotypes, three of them homozygous and three heterozygous, in nested ANOVAs for each gender. These analyses confirm that the MHC has a significant effect on parasitemia and blood cell counts (p = 0.009 and 0.003, respectively, Table 2) and on the time course of the parasitemia (p = 0.018, Table 2). However, the effects could only be observed in males. None of the MHC effects were statistically significant if tested in females only (Table 3).

**Table 2:** The effect of MHC type and MHC heterozygosity on the time course of disease symptoms in males.

	Parasitemia			Blood cell counts			Body weight change		
	F	d.f.	P	F	d.f.	P	F	d.f.	P
Between subjects									
MHC type	3.7	4, 79	0.009	4.4	4, 78	0.003	0.8	4, 78	0.53
MHC heterozygosity	0.2	1, 79	0.65	0.8	1, 78	0.39	0.1	1, 78	0.82
									
Within subjects (repeated measurements on individual mice)									
Time	121.2	5, 75	<0.0001	77.4	10, 69	<0.0001	21.7	18, 61	<0.0001
Time × MHC	1.8	20, 249.7	0.018	0.8	40, 263.5	0.77	1.0	72, 242.2	0.53
Time × heterozygosity	0.9	5, 75	0.48	1.0	10, 69	0.47	1.4	18, 61	0.15

Nested ANOVA incorporating the fixed effect factors "MHC-heterozygosity" and "MHC type" (nested in "heterozygosity"), with repeated measures each of the same disease symptoms as in Table 1. For within-subject analyses we used the multivariate F-tests or Wilk's lambda when a factor had more than two levels as in "MHC". The table combines our findings on the genotypes H-2^ak^, H-2^bk^, and H-2^kk ^with the data of Wedekind et al. [30] who used exactly the same methods on the host genotypes H-2^aa^, H-2^bb^, and H-2^ab^. This results in six MHC genotypes, three homozygous ones and three heterozygous ones. The analyses are separated for males (here) and females (Table 3) because the group H-2^kk ^was not represented in all experimental cells (a prerequisite for full-factorial analyses).

**Table 3:** The effect of MHC type and MHC heterozygosity on the time course of disease symptoms in females.

	Parasitemia			Blood cell counts			Body weight change		
	F	d.f.	P	F	d.f.	P	F	d.f.	P
Between subjects									
MHC type	1.5	4, 71	0.21	2.4	4, 71	0.62	0.4	4, 71	0.84
MHC heterozygosity	3.6	1, 71	0.061	0.7	1, 71	0.40	0.1	1, 71	0.69
									
Within subjects (repeated measurements on individual mice)									
Time	61.2	5, 67	<0.0001	107.2	10, 62	<0.0001	24.2	18, 254	<0.0001
Time × heterozygosity	0.7	20, 223.2	0.84	1.0	40, 237.0	0.45	1.0	72, 214.7	0.43
Time × MHC	1.5	5, 67	0.21	1.9	10, 62	0.065	0.5	18, 54	0.9

Nested ANOVA analogous to the one presented in Table 2

**Figure 2:**
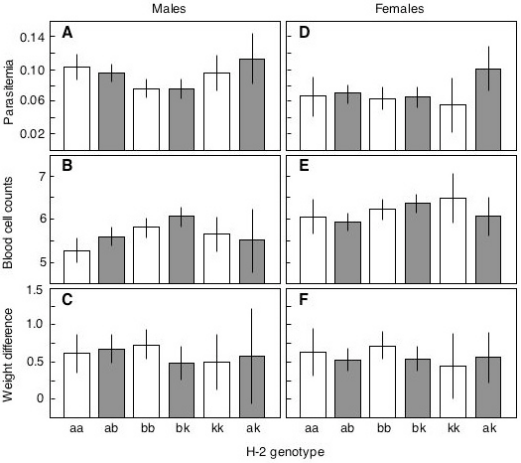
**The effects of six MHC genotypes on the average disease symptoms in males and females**. The figure combines previous results on H-2^aa^, H-2^ab^, and H-2^bb ^mice [30] with the new observations on H-2^bk^, H-2^kk^, and H-2^ak ^mice. The results (means ± 95% confidence intervals) are separated for male and female hosts and are the average per subject of 6 repeated parasitemia measurements from day 4–14, 11 counts of blood cell number (*10^9^)/ml blood from day 1–22, and 19 body weight measurements from day 4–22 given as difference to the weight at day 0 (in g). See Tables 2 and 3 for statistics (main effects of MHC-type).

Figure 2 gives the average (± 95% CI) disease symptoms of all six MHC genotypes we tested. It appears that none of the three heterozygous genotypes has lower parasitemia than any of their respective two homozygotes, i.e. we find no evidence for overdominance with respect to average parasitemia. Wedekind et al. [30] tested whether the heterozygous H-2^ab ^had higher parasitemia than what could be expected from the average of the respective homozygotes by first equalizing the sample size of the two homozygous variants before pooling them. This was done by randomly reducing the larger group to the size of the smaller group. The fully-factorial repeated measure ANOVA with the fixed factors heterozygosity, gender, and parasite clone was then calculated, and the whole procedure repeated 10 times to calculate an average F and p value (in Wedekind et al. [30] the heterozygous H-2^ab ^had higher parasitemia than expected from the average of the two homozygotes). Here we did the analogous analyses for H-2^ak^, comparing them to what would be expected from the homozygous H-2^a ^and H-2^k^, and for H-2^bk^, comparing them to what would be expected from the homozygous H-2^b ^and H-2^k^. None of the 2 × 10 runs resulted in a significant difference (H-2^ak^: F_1,18 _always < 3.0; H-2^bk^: F_1,48 _always < 2.4; p always > 0.10), i.e. we did not find any significant heterozygote advantage or disadvantage when we tested the two heterozygotes H-2^ak ^and H-2^bk^.

## Discussion

Most studies on the link between the MHC and a disease are based on correlative data with no experimental control for possible confounding effects of, for example, background genetics or non-genetic effects [2,34-37]. In the case of malaria, we know that the severity of the disease can be influenced by many genetic and non-genetic factors [1-12,35,38]. To elucidate the effects of particular MHC genotypes, it is therefore necessary to control for confounding factors and study experimental infections in an animal model. Here, we used an experimental design that controls for these potentially confounding effects. Our design allowed to test the separate and combined effects of various host and pathogen factors on the course of malaria in mice. We found that the MHC has a significant effect on the course of malaria. The H-2^ak ^genotype was more susceptible than the H-2^bk ^type. This finding is consistent with the previous observation that the homozygous H-2^a ^genotype confers greater susceptibility than the homozygous H-2^b ^when tested under the same conditions with F2s [30] and under less controlled conditions with the original congenic strains [13].

The severity of a disease can be seen as the result of an interaction between hosts and their parasites and not just as an intrinsic characteristic of a particular parasite clone [29,39,40]. The two parasite clones caused different disease patterns (confirming previous reports of their clone-specific characteristics [30,41,42]), but disease severity also depended on host characteristics: the two sexes react differently to the parasite clones (see the significant clone × gender interactions in Table 1). Some of these interactions were significant in a previous study [30], and are thus repeatable.

Our gender-specific analyses suggested that MHC effects on the course of malaria may be stronger in males than in females (Tables 2 and 3). Gender-specific virulence is common in vertebrates [43] and could potentially lead to more pronounced effects of host genes on pathogen susceptibility in the male sex [44,45]. However, possible gender-specific effects of MHC-specific disease patterns could not be confirmed in analyses that included gender as a factor, neither in the present study (Table 1) nor in a previous one [30], with the exception of a significant MHC × clone × gender effect on body weight change that was not expected [30]. Therefore, what appears to be a gender-specific MHC-link to malaria in Tables 2 and 3 could well be the consequence of some empty cells of the fully-factorial experimental design (that forced us to do gender-specific analyses), combined with gender-specific characteristics that are not linked to the MHC.

The peptide-binding motifs of MHC molecules bind only a small fraction of all peptides [46], i.e. different MHC molecules can be expected to bind largely to non-overlapping sets of peptides. This MHC specificity may well lead to significant parasite clone × MHC-genotype interactions and frequency-dependent selection [47-51], but our data could not support this. In particular, we found no significant interaction between parasite clone and MHC genotype for any of the disease symptoms in either the present study (MHC × clone, Table 1) or our previous one [30]. We suggest three mutually non-exclusive explanations for this non-significant finding: (i) The two clones, although clearly different in some respects, may be similar at some of their MHC-presented peptides, such that, for a given parasite clone, disease severity is relatively stable across host MHC types. (ii) Our statistical power may be too low to detect existing gene-for-gene interactions, and (iii) time- and/or condition-dependent protein expression may partly mask existing gene-for-gene interactions. The latter possibility seems to be supported by a significant three-way interaction (MHC × clone × gender on body weight change). Moreover, there was a non-significant tendency for blood cell counts to vary over time depending on the MHC × pathogen clone combination (p = 0.051, Table 1).

When fighting infection, MHC heterozygotes are often expected to be superior to both their respective homozygotes (i.e. exhibit overdominance) because they can present a wider range of antigens to T lymphocytes [52]. However, single infection experiments do not support this hypothesis [53]. In our study, too, MHC heterozygotes did not have significantly lower parasitemias than both the respective homozygotes. We had previously found that the heterozygotes H-2^ab ^did even worse than expected from the average response of the two respective homozygotes [30]. This finding could not be confirmed with the additional genotypes we studied here: heterozygotes performed neither better or worse than the homozygotes. Taken together, it appears that MHC heterozygosity has, on average, little or no effect on the course of malaria in single-strain infections.

## Conclusion

We found that variation in the MHC can have a significant effect on the course of a *Plasmodium *infection. The H-2^a ^haplotype is more susceptible than the H-2^b ^haplotype, regardless of whether its combined with H-2^k ^haplotype or tested in homozygotes, i.e. the previous findings [30] seem to be robust and repeatable. We could also confirm a significant effect of MHC-types on the course of parasitemia (the time × MHC effect in Table 1). Nearly all of the previously observed differences between the two parasite clones [30,41] could also be confirmed on the new host genotypes used here.

We have now compared three different MHC heterozygotes and their component homozygotes and found no heterozygote advantage in experimentally controlled single-clone infections. By contrast, under "field conditions", MHC heterozygotes may show slower disease progression and/or more rapid clearance of an infection than the average of homozygotes (e.g. [36,37]). MHC heterozygotes were indeed found to be overrepresented in some human populations [54,55], indicating a population-level advantage of heterozygosity that could, however, not be confirmed in other studies [56], and that cannot, for itself, explain the high degree of polymorphism of the MHC [57]. Moreover, even if a possible heterozygote advantage is observed in population studies, it is often unclear whether the advantage is due to overdominance, to dominance of resistance, or is explained only by the specific allele frequencies in a host population [58]. Haplotype-specific or allele-specific measures are therefore necessary to explain the kind of heterozygote advantage that is observed [53,59,60]. Our combined single-strain analyses suggests that either (i) parasites like *Plasmodium sp*. have specific characteristics (e.g. a relatively large antigen repertoire) that obscure any heterozygote advantages which would be expected in other kind of infections, (ii) heterozygote advantages are more frequent in multi-clone or multi-parasite infections than in single-clone infections, or (iii) heterozygote advantages are indeed less frequent than expected from previous studies, and the widely assumed and sometimes supported MHC-heterozygosity advantage in disease susceptibility is a rule with many exceptions.

Recent studies on pathogens with a presumably smaller antigen repertoire (Theiler's virus and Salmonella) confirm that MHC heterozygote advantage cannot generally be assumed in the case of single-clone infections (Penn *et al*. 2002; McClelland *et al*. 2003). In the case of *Plasmodium*, genetically variable infections are harder to clear and sometimes more virulent than single-clone infections [33,61], and thus may lead to different host-parasite interactions. Future studies might therefore incorporate a wider range of parasite clones, or might focus on the effects of multi-species or multiple-clone infections where the diversity of parasite antigens confronting the MHC could potentially change this result.

## Methods

### Mouse breeding and maintenance

Mice were housed in standard conditions at 21°C and on a 12:12 h light – dark cycle, with food provided *ad libitum *(41B, Harlan, UK) and 0.05% para-aminobenzoic acid added to their drinking water. We started with three MHC-congenic strains of mice (the MHC type given in parantheses): C57BL/10 (H-2^b^), B10.A (H-2^a^), and B10.BR (H-2^k^) obtained from B&K Universal, UK. In order to control for potential differences in the background mutation load and for possible differences in maternal effects, we crossed these three strains, reared the heterozygous offspring, and crossed them to produce the F2 generation that were used for present the experiments.

### Infections and monitoring

The two cloned *P. chabaudi *lines we used are denoted "AS" and "CW" [62]. Between isolation from the field and use in this experiment, the two lines had been blood passaged in C57Bl/6J (H-2^b^) mice for 8 and 6 times, respectively. These two clones were chosen for their relatively low growth rate and virulence compared to other clones [41].

Each mouse was infected *i.p. *to 10^5 ^parasites in 0.1 ml, prepared from donor mice by diluting blood in a calf-serum solution, i.e. 50% heat-inactivated calf-serum, 50% Ringer's solution (27 mM Kcl, 27 mM CaCl_2_, 0.15 M NaCl), 20 units heparin/ml of mouse blood. The parasites were obtained from one donor mouse each 6–7 days *p.i. *infected with frozen aliquots of blood. We infected the males first and the females three days later. Mean (± S.E.) age at infection was 47.5 (SE = 0.7 days). All experimental treatments of mice were performed in accordance with the Animal (Scientific Procedures) Act (1986) administered by the Home Office of the U.K. (licence no. 60/5640).

The mice were weighed to the nearest 10 mg on the day of infection and daily from days 4 to 22 *p.i*. Red blood cell densities were estimated by flow cytometry (Coulter Electronics) from a 1 : 40 000 dilution of a 2 μl sample of tail blood into Isoton solution on day 1 *p.i*., and every 2 days from day 4 to 22 *p.i*. Thin blood smears were made from tail blood every 2 days from day 4–14 *p.i*. These smears were stained with Giemsa. Parasitemia was determined as the average proportion of infected red blood cells. Therefore, the number of infected and uninfected red blood cells were determined in at least 10 randomly picked microscopic fields per blood smear (overall average = 124 red blood cells per microscopic field; only a quarter of the non-infected cells per field was counted if cell density was high). Parasitemia was determined from coded slides blindly with respect to genotype and experimental treatment. One mouse did not show signs of infection (no parasitemia) and was therefore excluded from the analyses. Infection was confirmed in all other mice.

### Genotyping

We used allelic variation at a microsatellite locus to determine the MHC genotype of each offspring produced in the crosses.Total genomic DNA was extracted (Amersham Biosciences Animal Tissue extraction kit) from tail samples that had been stored in 70% ethanol. Using oligonucleotide primers developed by Meagher and Potts [63] we initially examined variation at three loci which were likely to be sufficiently close to the MHC^d ^locus to permit discrimination of alleles. These loci were identified as 24, 34 and 148 [63]. Separately for each locus, DNA fragments were amplified via the polymerase chain reaction (PCR) using fluorescently labelled primers. Thermal cycling conditions were 25 cycles of 94°C denaturing, 50°C annealing and 72°C extension with times of 30 seconds each. A small quantity of each PCR reaction mixture was visualised on agarose gels to verify amplification. Allelic size variants were discriminated on an ABI 377 slab gel sequencer and the accompanying program Genescan 3.7 and Genotyper 2.5. Polymerase chain reactions successfully amplified ~120 to 150 bp DNA products for each of the loci tested. Only locus 34 proved sufficiently variable to discriminate all three size variants (alleles).

### Statistics

The experiment was designed for a fully-factorial repeated measures analysis of variance (ANOVA), with the factors host MHC, host gender, and parasite clone, and with repeated measure of a dependent variable each, i.e. parasitemia, blood cell density and weight change. Unfortunately, as the genotyping was only done after the exposure to the pathogens, some of the pathogen clone by sex combinations turned out to contain no replicates for the homozygous H-2^k^. We therefore used only the heterozygous H-2^ak ^and H-2^bk ^mice for the full model that includes gender and clone effects. The homozygous H-2^k ^mice could be included in gender-specific analyses, assuming that the pathogen clone × MHC interaction has no or negligible effects on disease symptoms (as was found in [30]).

All analyses were done with JMP 5.1 [64]. As the sphericity tests were each significant for the within-subject analyses, we used the multivariate F-tests (when factors had only two levels as in "gender" or "clone") or Wilk's lambda (when a factor had more than two levels as in "MHC").

## Abbreviations

MHC = major histocompatibility complex

PCR = polymerase chain reaction

## Authors' contributions

CW conceived the study, designed the experiment, participated in recording the disease symptoms and in the genotyping, did the statistical analyses, and wrote the first draft of the manuscript. MW optimized the parasitemia determination and analysed all blood samples. TJL carried out the molecular genetic analyses. All authors helped drafting the manuscript and approved the final version.
